# The Research Progress on Intestinal Stem Cells and Its Relationship with Intestinal Microbiota

**DOI:** 10.3389/fimmu.2017.00599

**Published:** 2017-05-23

**Authors:** Qihang Hou, Lulu Ye, Lulu Huang, Qinghua Yu

**Affiliations:** ^1^College of veterinary medicine, Nanjing Agricultural University, Nanjing, China

**Keywords:** intestinal stem cells, intestinal microbiota, niche, Paneth cell, intestinal epithelium

## Abstract

The intestine is home to trillions of microorganisms, and the vast diversity within this gut microbiota exists in a balanced state to protect the intestinal mucosal barrier. Research into the association of the intestinal microbiota with health and disease (including diet, nutrition, obesity, inflammatory bowel disease, and cancer) continues to expand, with the field advancing at a rapid rate. Intestinal stem cells (ISCs) are the fundamental component of the mucosal barrier; they undergo continuous proliferation to replace the epithelium, which is also intimately involved in intestinal diseases. The intestinal microbiota, such as *Lactobacillus*, communicates with ISCs both directly and indirectly to regulate the proliferation and differentiation of ISCs. Moreover, *Salmonella* infection significantly decreased the expression of intestinal stem cell markers Lgr5 and Bmi1. However, the detailed interaction of intestinal microbiota and ISCs are still unclear. This review considers the progress of research on the model and niches of ISCs, as well as the complex interplay between the gut microbiota and ISCs, which will be crucial for explaining the mechanisms of intestinal diseases related to imbalances in the intestinal microbiota and ISCs.

## Introduction

Inflammatory bowel disease (IBD), including Crohn’s disease (CD) and ulcerative colitis (UC), is the important cause of gastrointestinal disease (1). Although the precise etiology of IBD remains unclear and controversial, the intestinal microbiota and the integrity of mucosal epithelial function have been demonstrated to play key roles in its pathogenesis (2, 3). Moreover, diets high in fat and protein but low in fruits and vegetables have been demonstrated to be associated with particular compositions of intestinal microbiota that increase the risk of IBD (4). Moreover, a high-fat diet induces change of intestinal stem cells (ISCs) and enhances the risk for intestinal cancer incidence (5). Unlike other stem cells, ISCs coexist with the intestinal microbiota, which may influence the growth status of the epithelium (6). The intestinal mucosal barrier is maintained by ISCs, located at the base part of the intestinal crypts, play a key role in governing the proliferation and differentiation of the intestinal epithelium.

The gastrointestinal tract harbors a diverse community of microorganisms, including bacteria, fungi, and viruses, which are considered as the intestinal microbiota (7). The intestinal microbiota interact closely with ISCs. Although the elucidation of host pathways that regulate ISCs function is progressing, the effects of exogenous factors on ISCs biology are poorly understood. Recent studies demonstrated that ISCs protected itself from butyrate produced by beneficial microbes (8, 9). However, when the intestine tract is damaged, butyrate inhibits the proliferation of ISCs through preventing the intestine tissue from repairing itself after damage or injury (10). However, the detailed mechanism by which the intestinal microbiota regulates ISCs remains unknown. This review will focus on ISCs niches and the regulation of ISCs by the intestinal microbiota.

## Progress of Research on ISCs

**Figure 1:**
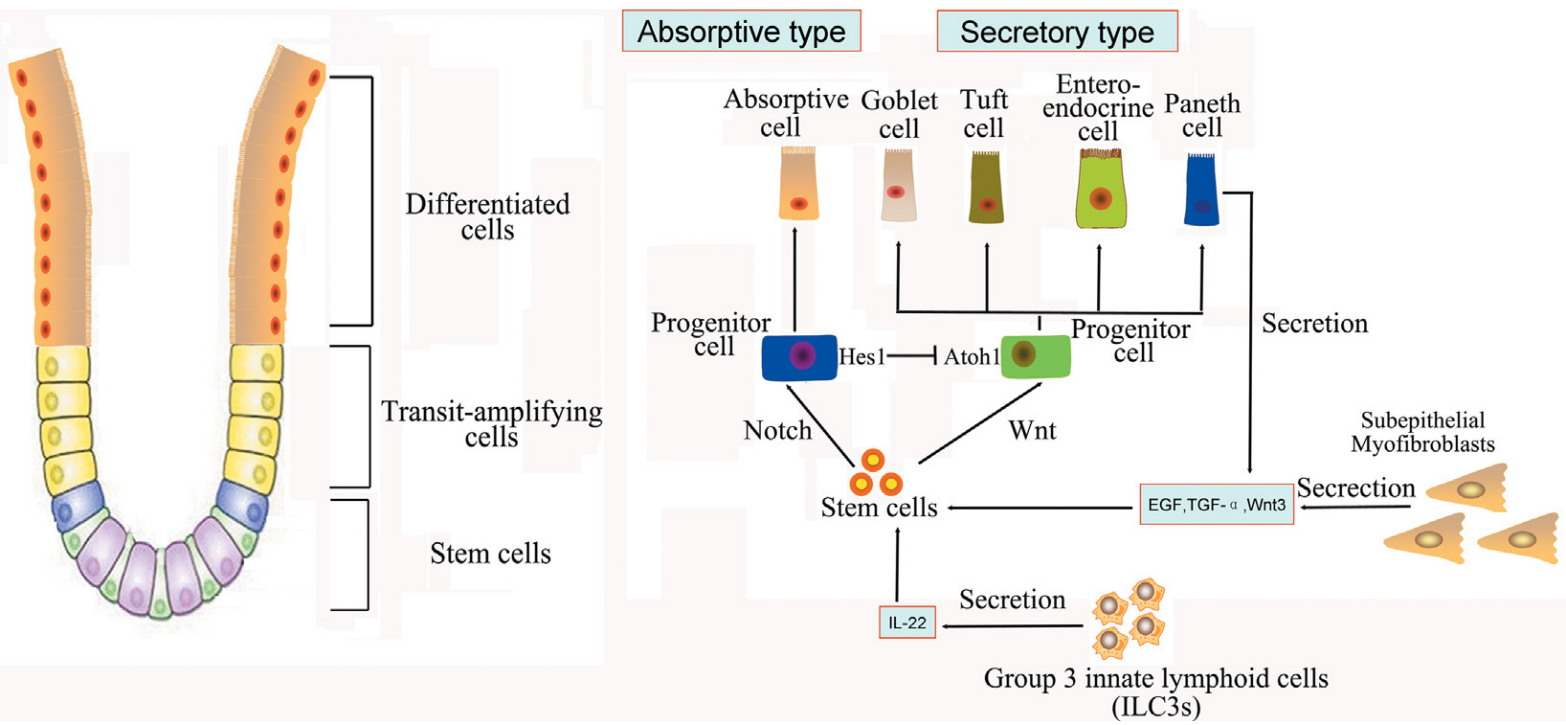
**Intestinal stem cells (ISCs) are periodically activated to produce progenitor or transit amplifying cells, which are committed to produce two mature cell lineages: absorptive type (enterocytes) and secretory type (enteroendocrine, goblet, tuft, and Paneth cells)**. The Paneth cells or subepithelial myofibroblasts could secret epidermal growth factor, TGF-α, Wnt3, and the Notch ligand Dll4, which are essential for the maintenance of ISCs, whereas their maturation depends on Wnt signaling. Innate lymphoid cells (ILC3s) could also activate ISCs regeneration through the secretion of IL-22.

The intestine is composed of columnar epithelial with glandular invaginations, and the intestinal columnar epithelium is continually shed and replaced by the self-renewal capacity of ISCs. Recently, Lgr5 was identified as an important active ISCs marker of ISCs located at the base of crypt, and Bmi1 was another marker of quiescent ISCs predominantly at the +4 position (11, 12). Detailed cell markers and functions of ISCs were listed in Table 1. Studies have shown that the Lgr + crypt base columnar cells (CBC cells) are rapidly dividing ISCs, which is necessary for gut renewal (13). Conversely, the +4 label-retaining cells (LRCs) are more quiescent, protecting them from the environmental stress. The +4 LRCs are activated during stress of injury, and subsequently produce intestinal progenitor cells to replace the damaged intestinal cells (14). Moreover, CBC cells can also regenerate new +4 LRCs under injury (15). Conversely, the active ISCs could replace the damaged quiescent stem cells under special conditions (15). The intestinal crypt maintains a balance between rapid-cycling, easy-to-damage stem cells, and quiescent +4 LRCs to maintain self-renewal and flexible damage repair (Figure 1).

**Table 1:** **Cell markers and functions of intestinal stem cells (ISCs)**.

Name	Functional description	Active vs. quiescent	Reference
Sox9	1 Transcription factor 2 The involvement of the proliferation and differentiation of embryonic stem/progenitor cells and CSCs through the Wnt/beta-catenin pathway	Active	(16, 17)
Ascl2	1 Basic helix loop helix transcription factor 2 The master regulatory gene for Lgr5 + ISCs 3 Overexpressed in intestinal neoplasia	Active	(18, 19)
MSI1	1 Translational repressor and involvement the Notch signaling 2 Control stemness in drosophila 3 Overexpression in intestinal epithelial progenitors enhancing their proliferative capacity	Active	(20, 21)
LGR5	1 R-spondin receptor 2 Wnt target and binding the R-spondin to enhance the downstream Wnt signaling 3 lgr5 also expressed in colorectal cancer	Active	(22, 23)
OLFM4	1 Encoding a secreted molecule with unknown function from human myeloblasts 2 Xenopus ONT1, an OLFM4 family member, acting as a BMP antagonist	Active	(24, 25)
Hopx	1 Atypical homeobox protein 2 Inhibit the Wnt signaling and maintain stem cell quiescence	Quiescent	(26, 27)
mTert	1 Enzymatic catalytic subunit of mouse telomerase 2 Targeting the TGF-beta pathway and increasing the proliferative potential of primary mouse embryonic fibroblasts	Quiescent	(28, 29)
BMI1	1 Polycomb transcription repressor complex 2 Transcription repressors 3 Part of the Polycomb group gene family, and specifically a member of polycomb-repressing complex 1	Quiescent	(30, 31)
DCLK1	1 Microtubule-associated protein kinase 2 DCAMKL-1 disruption resulting in inhibition of the Notch-1 pathway 3 Regulate the EMT through a miR-200a-dependent mechanism	Quiescent	(32, 33)

## The Establishment of ISCs Models

Studies using intestinal organoids are advancing our understanding of the role of the epithelium in intestinal physiology and pathophysiology. Intestinal organoids are composed only of epithelial cell types and are thus useful for understanding intestinal epithelial cell function in the absence of other cell types. For example, studies in mouse intestinal organoids have confirmed the importance of Paneth cells in the epithelial barrier at the intestinal organoid level. Paneth cells synthesize and secrete substantial quantities of antimicrobial peptides to modulate the homeostatic balance with colonizing microbiota and innate immune protection from enteric pathogens (34). Unexpectedly, epidermal growth factor (EGF), delta-like 1/4, and Wnt-3 are also expressed in Paneth cells, suggesting that this cell type provides ISCs niche signaling (35).

**Figure 2:**
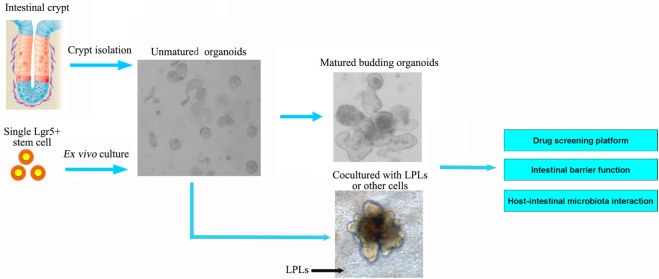
**Intestinal organoids, the *ex vivo* culture systems, are ideal intestinal structure models to explore the interaction between intestinal microbiota and epithelial cells, as well as for drug screening and intestinal barrier function**. The organoids could be established from the isolated crypt from the intestine or single intestinal stem cell. The current models of intestinal organoids lack enteric nerves and immune cells. In future, the dendritic cells or lamina propria lymphocytes (LPLs) could be cocultured *in vitro* to improve the model further.

The new culture condition established by Wang et al. could produce highly homogeneous population of ISCs, avoiding heterogeneous cell populations in 3D organoids *in vivo* (36). Current culture method for ISCs relies on a 3D culture system using Matrige, which is not approved by FDA for clinical use. A new 2D culture system for expansion of ISCs as an intestinal epithelial monolayer and cultured on a thin coat of type I collagen or laminin instead of Matrigel was established (37). Moreover, current models of intestinal organoids lack enteric nerves and immune cells, and one goal is to add these components to develop a more complex *in vitro* intestinal model using coculture approaches. Long-term culture of the human small intestine epithelium requires the presence of subepithelial myofibroblasts, even when exposed to Wnt3a-containing media (38). Most recently, reports have demonstrated the successful regeneration of the human small intestine from collagenase-digested “organoid units” that contain Lgr5 + ISCs and mesenchyme. Group 3 innate lymphoid cells (ILC3s) have also been shown to be important for maintaining ISCs proliferation (39) (Figure 2).

The intestinal organoids containing ISCs is a promising model to explore the interaction between intestinal microbiota and intestinal mucosa. *Salmonella* could infect the intestinal organoids, and the infection also significantly decreased the expression of intestinal stem cell marker Lgr5 and Bmi 1 (40, 41). Intestinal organoids could not only be infected by rotavirus, and infected organoids are also capable of producing infectious rotavirus particles (42, 43). The human noroviruses (HuNoVs) have been successfully cultivated in enterocytes in stem cell-derived, non-transformed human intestinal enteroid monolayer cultures, and the Replication of HuNoV Replication occurred in a bile dose-dependent manner (44).

## Research Regarding the ISCs Niche

The activity of ISCs is tightly regulated by several niche-signaling pathways to balance the intestinal homeostasis under physiological and pathological stimulation. The Wnt signaling has been identified as the most indispensable pathways for ISCs. Recently, study demonstrated that Wnt3 produced specifically by Paneth cells shapes the concentration gradient in the intestinal crypt, and the ISCs membrane constitutes a reservoir for Wnt proteins (45). R-spondin-1 cooperates with Wnt3 pathway activation through the modulation of Rnf43 (46). Furthermore, ASCL2 regulated downstream of the Wnt pathway has also been shown to be ISCs-specific genes (47).

Besides Wnt signaling, the Notch pathway is another indispensable pathway in regulating the proliferation and differentiation of ISCs (48). Notch1 and Notch2 are two indispensable receptors for maintaining the normal proliferation and differentiation of ISCs in the intestine (49, 50). Loss of the ligands Dll1 and Dll4 could induce the silencing of Notch activation in intestinal epithelial cells (51). Activation of Notch pathway could stimulate ISCs differentiation into absorptive cell lineages, while cis-inhibition of Notch directs ISCs toward secretory lineage cells, such as goblet cells, enteroendocrine cells, Paneth cells, or tuft cells (48). Recent *in vitro* and *in vivo* models have suggested that Notch suppression reduces the ratio of BMI1+/LGR5+ ISCs, while Notch stimulation increases this ratio. Furthermore, Notch signaling can activate asymmetric division after intestinal inflammation (52).

BMP signaling is also required for the maintenance of ISCs replication and the terminal differentiation of intestinal cells. Both epithelial and mesenchymal cells could produce BMP ligands. BMP signaling can suppress Wnt signaling to ensure the appropriate balance of epithelial stem cell self-renewal (53). BMP signaling controls the terminal differentiation of the intestinal secretory cell lineage (51). However, BMP2 inhibits epithelial cell growth in the colon by promoting apoptosis and differentiation (52). Several other signal pathways, such as EGF and Hippo signaling pathway also take part in the formation of ISCs niches (54).

## Intestinal Mucosa Injury with Intestinal Microbiota Dysbiosis

In the physiological state, the intestinal microbiota either has direct bactericidal effects or inhibits the adherence and invasion of pathogens to the gut mucosa (55). However, intestinal microbiota dysbiosis may facilitate the adhesion of pathogens that may be associated with irritable bowel syndrome (IBS) symptoms (56). The exact cause of IBS is also unknown and is thought to be multifactorial. Variation in the gut microbiota is thought to be complicit in the low-grade intestinal inflammation associated with this syndrome (57). Alterations in the microbiota composition in IBS patients may aggravate the development of IBS symptoms (58). The relative abundance of the *Firmicutes* population may be greatly reduced in IBD patients, which is of particular interest because these bacteria are known producers of important short-chain fatty acids, which have potent anti-inflammatory properties (59). The defects in ISCs differentiation to Paneth cells or goblet cells are always observed in IBD, which results in luminal microbe invasion of the mucosa and inflammation (60).

Inoculation of UC patients with feces from healthy population, who were extensively screened for parasites and bacterial pathogens, relieved the severe and recurrent inflammatory symptoms within 1 week, and induce complete reversal of inflammatory symptoms in 4 months after the fecal transfer in UC patients, which indicated that infusion of healthy donor human intestinal flora can reverse UC (61). The promise of intestinal stem cell biology lies in the ability of these remarkable cells to give rise to more differentiated intestinal cell types that can repair damaged or diseased tissues. Several therapeutic approaches, including intestinal organoids, are currently being explored as a possible treatment for intestinal disease (62–64). Moreover, recent studies also demonstrated that gut–microbe interactions are also involved in determination of ISCs activity through Janus kinase–signal transducers and activators of transcription (JAK–STAT) pathway, which indicated modulation intestinal microbiota could also stimulate the ISCs proliferation to treat intestinal injury (65–67).

## Effects of the Intestinal Microbiota on ISCs

### Regulation of the Intestinal Microbiota by Wnt and Notch Signals through Pattern Recognition Receptors (PRRs)

The intestinal epithelium recognizes bacterial components through PRRs and communicates with the resident luminal bacteria (68). The innate immune system senses the pathogenic invasion or epithelium injury *via* toll-like receptors (TLRs) and nod-like receptors and provide immediate responses (69). Recent evidence suggests the existence of cross talk among the Wnt and Notch pathways, TLR signaling, and the microbiota (70). Previous study demonstrated that, in alveolar epithelial cells, the Wnt/β-catenin is a negative feedback loop to repress TLR-triggered inflammatory responses (71). TLR signaling has been shown to alter intestinal homeostasis and to affect the proliferation and apoptosis rates in the crypt (72). It has been shown that ISCs also express TLR4, and the direct activation of TLR4 on ISCs, especially Lgr5-positive ISCs, regulates their ability to proliferate in intestinal crypts. TLR4 suppressed Wnt signaling, decreased activation of the Wnt receptor LRP6, and blocked the protective effect of the Wnt3a ligand (73). Epithelial differentiation into goblet cells was increased upon inhibiting the Notch signaling in the intestine, and the Notch signaling could also be modulated by TLR4 (74, 75).

Within the intestinal crypt, Lgr5+ stem cells constitutively express much higher levels of the cytosolic innate immune sensor Nod2 than do Paneth cells (76). Stimulation of Nod2 by its bona fide agonist, muramyl dipeptide, a peptidoglycan motif common to all bacteria, triggers stem cell survival, which leads to strong cytoprotection against oxidative stress-mediated cell death. Thus, gut epithelium restitution is Nod2 dependent and triggered by the presence of microbiota-derived molecules (76).

### The Effect of Reactive Oxygen Species (ROS) Produced by the Intestinal Microbiota on the Regulation of ISCs

**Figure 3:**
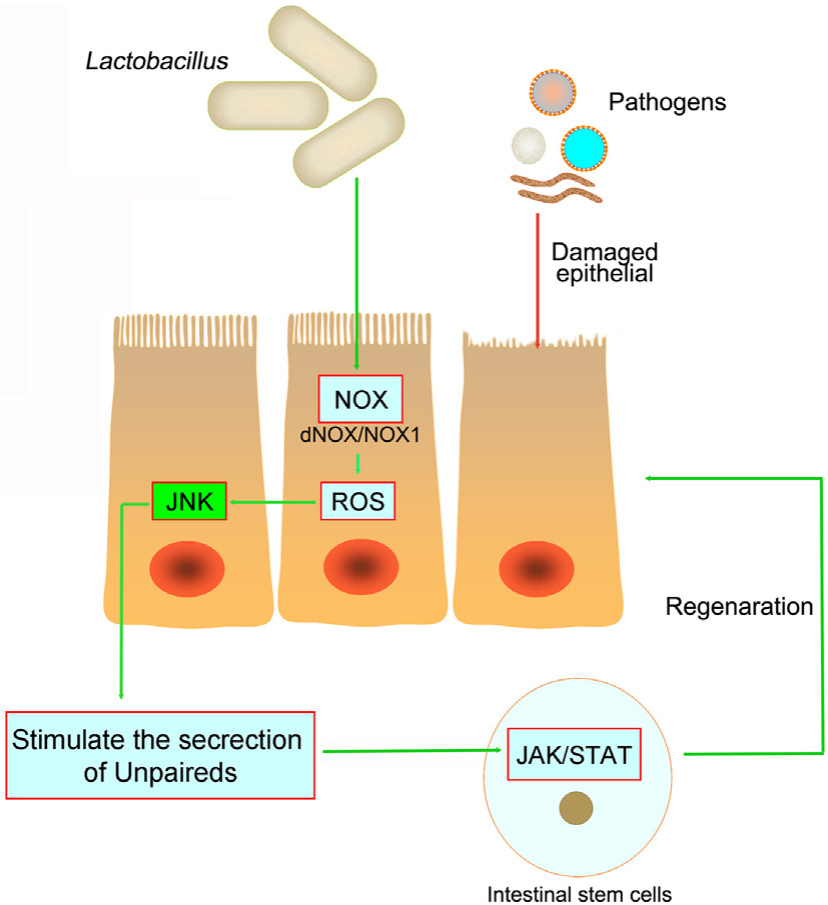
**The pathogens in the intestine damage the intestinal epithelial and cause inflammation**. However, both in *Drosophila* and mice, *Lactobacillus* could stimulate the intestinal epithelial to produce reactive oxygen species (ROS), and then stimulate JNK signaling and increase unpaired expression, thereby stimulating intestinal stem cells proliferation and epithelial turnover or regeneration.

The interaction between gut and intestinal microbiota is critical for the ISCs proliferation and differentiation, as well as the modulation of epithelium regeneration. However, the detailed mechanism of ISCs activation after microbe-induced issue damage is currently unknown. ROS, traditionally viewed as toxic, is now clearly recognized as a key modulator in all kinds of biological processes. Recent study has demonstrated that intestinal epithelia contacted by enteric commensal bacteria rapidly generate ROS, and physiologically generated ROS acts as signaling molecules to mediate increased cellular proliferation and motility and to modulate innate immune signaling (77, 78) (Figure 3). However, high levels of ROS during enteric infections likely act indiscriminately against both commensals and pathogens (65, 79). *S. Typhimurium* infects and damages the intestinal epithelial, then promotes migration of neutrophils that produce ROS, which facilitate conversion of S_2_O_3_^2−^ generated by commensal bacteria, into S_4_O_6_^2−^ (80, 81). Commensal *Lactobacilli* stimulates ROS production *via* Nox rather than Duox, thereby activating ISCs in Drosophila and mice under physiological status (82, 83). Recent studies demonstrated that redox homeostasis is critical in the regulation of stem cell differentiation and ROS specifically modulate the stem cell self-renewal (84). Many molecules involved in ROS-regulated stem cell self-renewal were redox sensors, which were found to be modified at redox-active cysteine residues (78). The Wnt and Notch signaling pathways can also be affected by ROS, which then influence the proliferation of ISCs (85). However, we are still not clear whether ROS act as direct inducers of ISCs signaling or simply cause epithelial damage that signals to ISCs.

### The Effect of the Intestinal Microbiota on Paneth Cells

Paneth cells, located at the base of the crypts in the small intestine, are highly specialized secretory cells. As an important source of antimicrobial peptides, Paneth cells have an antimicrobial function and regulate the intestinal microbiome by secreting bactericidal proteins such as α-defensins and lysozyme (34). *Salmonella* infection could stimulate the expansion of the Paneth cells population with increased expression of MyD88 in Paneth cells, which is sufficient to limit *Salmonella* penetration across the mucosal barrier (86, 87).

Recent study also demonstrated that Paneth cells are important for the differentiation and proliferation of ISCs, which are interspersed between Paneth cells (35). Paneth cells secret EGF, TGF-α, Wnt3, and the Notch ligand Dll4, which are essential for the maintenance of ISCs, whereas their maturation depends on Wnt signaling (88). Coculturing of ISCs with Paneth cells could markedly stimulate the formation of intestinal organoids *in vitro* (35). Crypt cells do not grow *ex vivo* after the inducible deletion of transcription factor Math1 (Atoh1). However, the complete loss of Paneth cells did not damage the intestinal crypt structure and maintain the physiological proliferation of ISCs *in vivo*, which implied the underlying mucosal cells may act as a potential ISCs niche (89).

Paneth cells are an initial source of IL-1β signaling during early infection with pathogens, causing gut inflammation. However, intestinal inflammation can be controlled by treatment with *Lactobacillus plantarum via* the reversal of IL-1β signaling (90). Moreover, the release of antimicrobial products by Paneth cells was controlled by IFN-γ (91). Recently, Paneth cells could augment stem cell function in response to caloric restriction. Calorie intake could regulate the mTORC1 pathway in Paneth cells and affect the ISCs niche (92). Paneth cells are able to directly sense commensal gut bacteria. The role of Paneth cells as critical mediators of microbe-ISC interactions is critical for ISCs, and this topic deserves further exploration.

### The Relationship between Crypt-Specific Core Microbiota (CSCM) and ISCs

**Figure 4:**
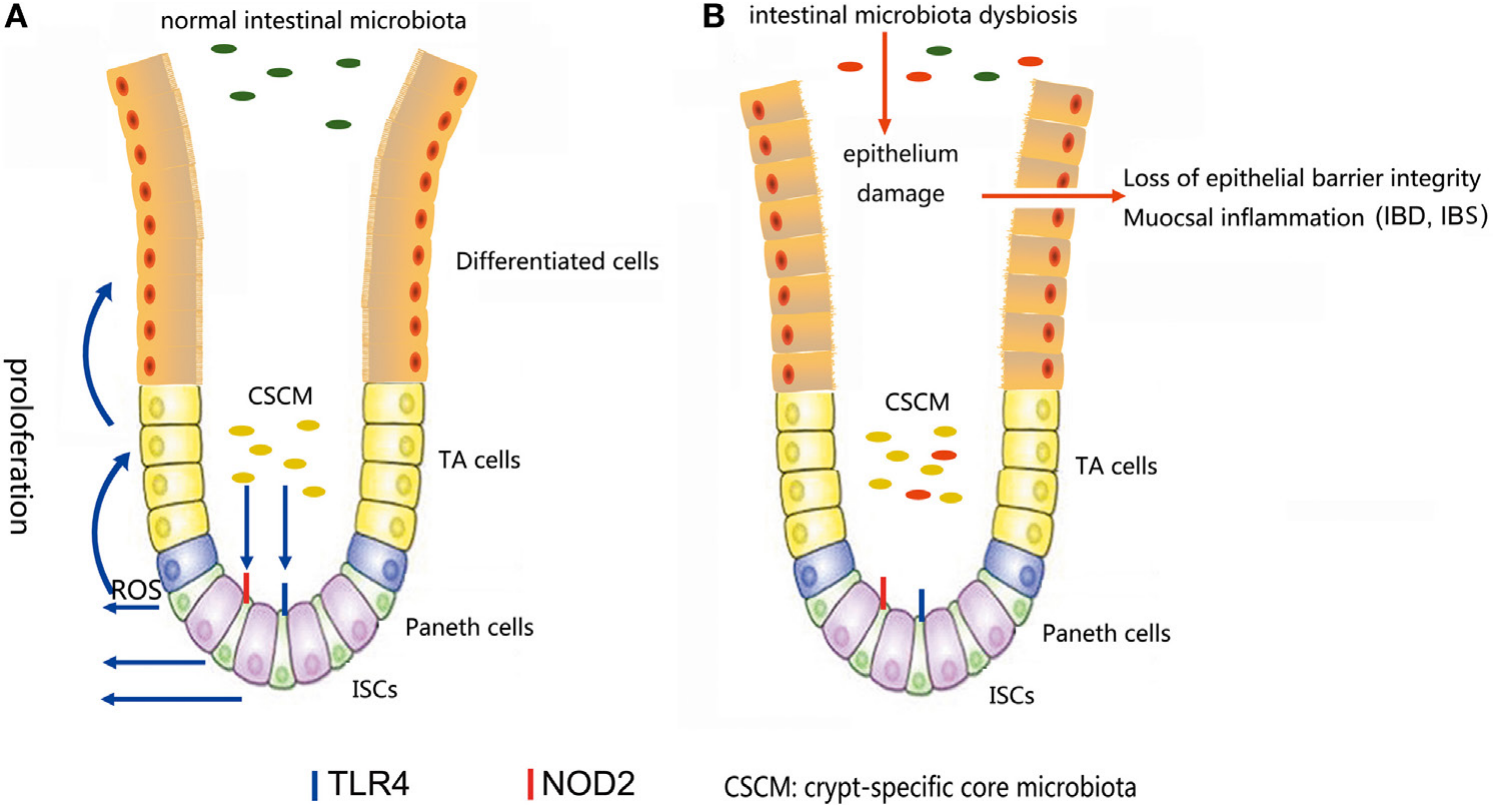
**(A)** In a normal intestinal microbiota environment, the microbiota, especially the crypt-specific core microbiota (CSCM), survive in the crypt, where they can stimulate the proliferation of intestinal stem cells (ISCs) and induce their differentiation to repair the intestinal epithelium *via* the TLR4, NOD2, or reactive oxygen species (ROS) signaling pathway to maintain the integrity of the mucosal barrier. **(B)** Under the pressure of microbiota dysbiosis, the composition of the microbiota and the CSCM will change and affect the stem cell niches, thus hindering the physiological processes regulated by stem cells, inducing epithelial damage and causing mucosal inflammation.

The microbiota provides continuous stimulation to the intestinal epithelial and affects the ISCs differentiation and proliferation. Recent studies further demonstrated that several signals induced by intestine–microbe interactions are involved in the determination of ISCs activity (93). A particular CSCM was found to survive in the colonic crypt environment (93). The CSCM could prevent the proliferation intestinal pathogens and provide optimal signaling to ISCs (Figure 4). Several particular species, such as *Acinetobacter* genus, may be evolutionarily selected because they provide advantage to the intestinal epithelial, probably through the expression of particular microbe-associated molecular patterns or the production of specific metabolites to maintain crypt homeostasis (6).

### The ISCs Development under Germ-free Conditions

Mammalian intestines are similar in structure and function with Drosophila, but the gut microbiota of Drosophila is composed of only 5–10 bacterial species, thus making it an ideal model to explore the intestine–microbe interactions (94, 95). Comparative studies of germ-free and conventionally reared Drosophila demonstrated that intestinal microbiota alters intestinal homeostasis, intestinal metabolism, and digestive enzymes expression (95, 96). Furthermore, the intestinal microbiota affects the ISCs activity by the modulation of nutrition metabolism. The damaging effects of a lack of microbiota are more evident in old flies, as the intestinal mucosa of germ-free flies was less over-proliferation and misdifferentiation than conventionally reared flies (97).

In Drosophila larvae, the intestinal microbiota promotes growth in nutrient-scarce conditions, and the intestinal peptidase expression and proteolytic activity were recovered after re-association with *L. plantarum* alone, thus leading to increased digestive abilities (98). *Lactobacilli* modulate ISCs and stimulate gut epithelium proliferation *via* the Nox-mediated generation of ROS (79, 83). The intestinal microbiota can become imbalanced after an injury, and probiotics can help the gut flora to recover and restore balance in the gut (99). Unlike the normal microbiota, *Pseudomonas entomophila* and *P. aeruginosa* cause the epithelial cell loss in the midgut and induce apoptosis in intestinal epithelial cells, resulting in ISCs over-proliferation (100).

The intestinal microbiota promotes substantial changes in intestinal morphology, including villus structure, crypt depth, stem cell proliferation, and maturation of mucosa-associated lymphoid tissues. In the absence of bacteria, intestinal crypts are less deep and contain fewer proliferating stem cells (101). During the suckling period in mice, extensive dynamic epigenetic changes are observed in ISCs, and the postnatal DNA methylation increased at 3′ CpG islands (CGIs) are responsible for intestinal maturation. Moreover, the DNA methyltransferases, *Dnmt1* is a critical regulator of postnatal epigenetic changes in ISCs. However, the postnatal 3′ CGI methylation and associated gene activation in ISCs are significantly altered by germ-free conditions (102).

## Future Possibilities

Most recently, IL-22 was shown to be important for maintaining the proliferation of ISCs. Previous studies have demonstrated that Paneth cells or intestinal subepithelial myofibroblasts can secrete Wnt and R-spondin-1 to regulate ISCs function and induce epithelium regeneration. However, the most recent reports have shown that the immune system can support the intestinal epithelium, activating ISCs to promote regeneration through IL-22 secretion by ILC3s (39). Furthermore, the intestinal microbiota can modulate the mucosal immune response, including the secretion of IL-22. However, whether the intestinal microbiota can stimulate ISCs proliferation through IL-22 secretion remains unknown. This phenomenon opens up the possibility of exploring the interaction between the intestinal microbiota and ISCs.

Intestinal organoids containing ISCs transplantation is a promising therapy method for cure intestinal inflammation. Single Lgr5 + ISC-derived colonoids transplantation accelerated the recovery of epithelial barrier function and reversal of inflammation in the DSS colitis model (103). The regulation effects of intestinal microbiota on ISCs need to be further explored for future application of intestinal microbiota to prevent intestinal inflammation. Several challenges, such as the difference between rodent model and human systems, efficient protocol to enrich ISCs, ensuring the safety and efficacy of ISCs-based products, need to be solved in the further.

## Author Contributions

QH and LY contributed equally, responsible for the literature search, figures, study design, and data collection; LH is responsible for data analysis of table design; QY is responsible for the conception and design of the study, drafting the article, and final approval of the version to be submitted.

## Conflict of Interest Statement

There are no potential conflicts (financial, professional, or personal) that are relevant to the manuscript.
